# Infection prevention and control of *Clostridium difficile:* a global review of guidelines, strategies, and recommendations

**DOI:** 10.7189/jogh.06.020410

**Published:** 2016-12

**Authors:** Evelyn Balsells, Teodora Filipescu, Moe H. Kyaw, Camilla Wiuff, Harry Campbell, Harish Nair

**Affiliations:** 1 Centre for Global Health Research, Usher Institute of Population Health Sciences and Informatics, University of Edinburgh, Edinburgh, Scotland (UK); 2 Sanofi Pasteur, Swiftwater, PA, USA; 3 Health Protection Scotland, Scotland, UK; 4 Public Health Foundation of India, New Delhi, India; * Joint last authorship

## Abstract

**Background:**

*Clostridium difficile* is the leading cause of health care–associated infections. Given the high incidence of *C. difficile* infection (CDI) and the lack of primary prevention through immunization, health care professionals should be aware of the most current guidance, as well as strengths and limitations of the evidence base underpinning this guidance.

**Methods:**

We identified publicly available national or organizational guidelines related to CDI infection and prevention control (IPC) published between 2000 and 2015 and for any health care setting through an internet search using the Google search engine. We reviewed CDI–targeted IPC recommendations and describe the assessment of evidence in available guidelines.

**Results:**

We identified documents from 28 countries/territories, mainly from acute care hospitals in North America, the Western Pacific, and Europe (18 countries). We identified only a few specific recommendations for long–term care facilities (LTCFs) and from countries in South America (Uruguay and Chile), South East Asia (Thailand), and none for Africa or Eastern Mediterranean. Of 10 IPC areas, antimicrobial stewardship was universally recognized as essential and supported by high quality evidence. Five other widely reported “strong” recommendations were: effective environment cleaning (including medical equipment), case isolation, use of personal protective equipment, surveillance, and education. Several unresolved and emerging issues were documented and currently available evidence was classified mainly as of mixed quality.

**Conclusion:**

Our review underlines the need for targeted CDI IPC guidelines in several countries and for LTCFs. International harmonisation on the assessment of the evidence for best practices is needed as well as more robust evidence to support targeted recommendations.

*C. difficile* is the leading cause of health care–associated infections (HAI) worldwide affecting especially the elderly and hospitalised patients [1–5]. The burden of CDI remains under–recognized and challenges associated with case detection hinder prevention. It was estimated that in 2011, over 450 000 CDI cases occurred in the United States and 172 000 in Europe [6,7]. Mounting evidence of the rising importance of CDI in other regions, such as Asia [8,9] and Latin America [10,11] contributes to concerns about the wide–ranging reach of CDI morbidity [6,12,13]. Given the high incidence of CDI and the lack of primary prevention through immunization, health care professionals should be aware of the most current guidance, as well as strengths and limitations of the evidence base underpinning this guidance.

There are wide variations in the availability or levels of implementation of effective Infection Prevention and Control (IPC) measures for CDI. A national survey in Canada identified an extensive lack of antimicrobial stewardship programmes, less than 25% of the 33 participating hospitals [14] in 2005. More recently, attention was drawn to the lack of clinical awareness and testing [15], disparities in the strength of recommendations across different IPC guidelines [16], and the lack of knowledge on the independent effects of common IPC strategies [17–19]. As guidelines are useful tools to promote coordinated IPC efforts, a detailed documentation of current published strategies has the potential to highlight commonalities and discrepancies in recommended practices. A comprehensive overview of published guidelines also has the potential to inform the decision–making of infection control stakeholders at the national, provincial, and institutional level and help researchers in targeting current gaps in the literature.

In this review, we describe the availability of documents that outline recommendations and actions for the prevention and control of CDI. We present a structured assessment of key elements of CDI–IPC strategies together with their strengths of recommendation and levels of evidence across 10 IPC areas followed by a discussion of current issues. A summary of unresolved issues to inform future research is also provided.

## Search strategy and selection process

Two reviewers (EB, TF) conducted an internet search (with the Google search engine) in July 2015 of publicly available national or organizational guidelines, related to CDI control (published between 2000 and 2015 and for any health care setting). Keywords used included “*difficile*” “*clostridium* *difficile*”, “policy”, “strategies”, “control”, “prevention”, “recommendation”, “guideline”, and “protocol.” Guidelines were defined as documents with systematically developed statements to assist practitioners and patients to make decisions about appropriate health care for specific clinical circumstances [20] or documents guidance from professional entities, which described IPC guidance and strategies for CDI. We retrieved the most updated and/or comprehensive documents principally from national departments/ministries of health and the websites of professional societies including those members of the International Federation of Infection Control. No language restrictions were applied. GoogleTranslate was used as the main translation tool for documents in languages other than English, Spanish, and Romanian (which were read directly by reviewers). Manuals containing generic HAI guidelines and documents with guidelines for treatment or policies of individual hospitals were not included. Structured abstraction of the recommendations from guidelines was conducted independently by the two reviewers and compared for 10 areas relevant to CDI–IPC, drawing from previous work [16,21,22].

## Presentation of results

For each area, we first present a brief description of the guidance identified, followed by a summary of the quality of evidence assessment and strength of recommendations identified in the guidelines (see below). We then present a discussion of current literature supporting recommendations or an overview of relevant issues.

## Quality of evidence and strength of recommendations

Seven documents graded the quality of evidence [23–29] (four ranking systems used) and nine provided strength of recommendations [23,24,27–33] (five ranking systems). The data quality categories of the ranking systems were broadly similar, and were grouped in three descriptive categories (high, medium, and low). The strength of recommendations for implementation were grouped into the following categories: *strong recommendation* (two levels differentiated by quality of supporting evidence); *recommended*, *consideration,* and *legal requirement.* Strategies were also classified as *Basic, Special* (ie, likely to reduce risk but concerns exist about undesirable outcomes), or *Unresolved Issue*/*Area of Research/Inconclusive* in one guideline [26]. (See Appendix S2 in **Online Supplementary Document(Online Supplementary Document)**).

## RESULTS

### Availability of guidance for CDI–IPC

**Figure 1:**
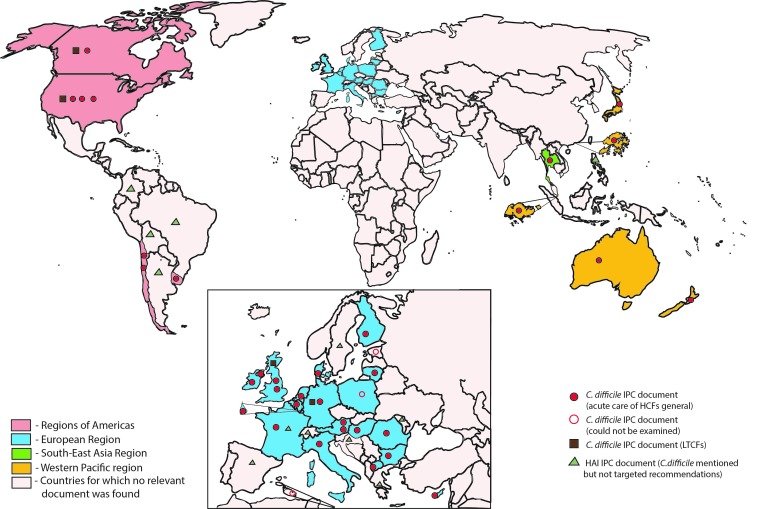
Geographic distribution of CDI-targeted IPC guidance reviewed. Countries with documents included in the review are colored by WHO regions (see legend left). Regional guidance by professional organizations (ECDC and ASID) are not depicted. Other results from the web–based search are also shown with different symbols (see legend right). Documents from countries with empty red circles were not examined as could not be translated (Poland) or full text could not be obtained (Malta, Latvia) but have previously been assessed [16].

Globally, 42 documents with targeted IPC recommendations for CDI were identified (**Figure 1**). These documents described guidance from 28 different countries/territories in 4 WHO regions. A summary of the main characteristics of these documents is available in Appendix S1 in **Online Supplementary Document(Online Supplementary Document)**.

In North America, 2 Canadian government advisory documents [34,35] and 4 documents from US–based professional bodies (3 guidelines [23,26,27], and an implementation guide [36]) were identified. In Europe, documents from government and professional organizations from 18 countries [24,25,28,30–33,37–54] and by the European Centre for Disease Control (ECDC) [29] were reviewed. Eleven guidelines reported grading for either the quality of evidence or strength of recommendation for implementation in their statements [23–33]. In the Western Pacific region, descriptive advisory reviews of guidelines by governmental agencies [55–58] and two professional groups (Australasian Society for Infectious Disease (ASID)/Australian Infection Control Association (AICA)) were included [59,60]. In South America, government guidelines from Chile [61,62] and Uruguay (draft) [63] were identified. In South East Asia, a document by a Thai professional organization which combines a review of the literature with a short section (6 items) on the prevention of CDI [64] was identified. No documents were identified from the Eastern Mediterranean or Africa regions.

### CDI–IPC strategies in non–acute care facilities

No specific recommendations were identified for CDI patients in skilled–nursing facilities, such as residential care and nursing homes, outpatient care, rehabilitation, and long–term care facilities (LTCFs). *C. difficile*–targeted IPC strategies mainly drew from evidence from acute care settings. Four guidance documents were specific to LTCFs and in other nine, recommended strategies were combined with guidance for acute hospitals. Relevant issues and challenges for the prevention of CDI in LTCFs were highlighted including: the vulnerable health status of residents which may pose difficulties in maintaining precautions (eg, cognitively impaired patients [58], frequent stool incontinence [36]); the placement of CDI cases in LTCFs in shared rooms due the limited number of single rooms [36]; and the lack of convenient hand–washing facilities [27,35]. The importance of surveillance, monitoring of outbreaks, and communication between ambulance services and staff in acute care facilities (when residents with CDI needed to be transported) was discussed [58], especially in the light of the under–recognized burden of CDI and imperfect adherence to IPC guidelines in LTCFs (including private and voluntary nursing homes) [25,31].

### Recommended strategies within IPC areas

Approaches to reduce transmission and to minimise host susceptibility by prudent antibiotic use were widely reported, but differences in other areas existed, as shown in **Table 1**, **Table 2** and **Table 3**.

**Table 1:** Overview of selected IPC strategies in health care facilities in guidelines and documents reviewed, by IPC area*

	Canada	USA	ECDC	Austria	Belgium	Bulgaria	Cyprus	Denmark	Finland	France	Germany	Hungary	Ireland	Italy	Lithuania	Luxembourg	Macedonia	Netherlands	Romania	UK-England	UK-N. Ireland	UK-Scotland	ASID (a/AICA)	Hong Kong	Japan	New Zealand	Singapore	Thailand	Chile	Uruguay	USA	Canada	Germany
**Pharmacological methods**:																																	
Antibiotic stewardship	✓	✓	✓	✓	✓	✓	✓			✓	✓	✓	✓	✓	✓	✓	✓	✓	✓	✓	✓	✓	✓	✓	✓	**†**	**†**	✓	✓	✓	✓	✓	
Probiotics		Inc^a^, UI^b^ RNM^c^	RNM	**†**^d^			RNM					**†**	**†**	RNM			**†**		**†**	RNM^f^		**†**	RNM^h^	AR	**†**			**†**			RNM		
Decrease use of PPI, H2RA		**†**^a^, UI^b^					**†**						**†**							✓^e,f^		**†**	**†**							✓			
Vaccines or immunotherapy		**†**^c^																		**†**		**†**											
**Contact precautions:**																																	
Isolation room	✓	✓	✓	✓	✓	✓	✓	✓	✓	✓	✓	✓	✓	✓	✓	✓	✓	✓	✓	✓	✓	✓	✓	✓	✓	✓	✓		✓	✓	✓	✓	✓
Cohorting	✓	✓	✓	✓	✓	✓	✓	✓	✓	✓	✓	✓	✓	✓	✓		✓	✓ ^§^	✓	✓		✓	✓	✓		✓	✓		✓	✓	✓	✓	✓
Duration precautions	✓	✓	✓	✓	✓	✓	✓	✓	✓	✓	✓	✓	✓	✓	✓	✓		✓	✓	✓	✓	✓	✓^h^	✓		✓	✓		✓	✓	✓	✓	✓
Decrease in case movement (transfers)	✓	✓		✓	✓	✓	✓	✓		✓	✓	✓	✓		✓					✓	✓	✓	✓			✓				✓		✓	✓
**Personal protective equipment:**																																	
Gloves	✓	✓	✓	✓	✓	✓	✓	✓	✓	✓	✓	✓	✓	✓	✓	✓	✓	✓	✓	✓	✓	✓	✓	✓	✓	✓	✓	✓	✓	✓	✓	✓	✓
Gowns	✓	✓	✓	✓	✓	✓	✓	✓	✓	✓	✓	✓	✓	✓	✓	✓	✓	✓	✓	✓	✓	✓	✓	✓	✓	✓	✓		✓	✓	✓	✓	✓
Hand hygiene:	✓	✓	✓	✓	✓	✓	✓	✓	✓	✓	✓	✓	✓	✓	✓	✓	✓	✓	✓	✓	✓	✓	✓	✓	✓	✓	✓	✓	✓	✓	✓	✓	✓
Wipes (W) Aseptic soap (AS)	✓W	**†**^a^	AS:UI							AS:AR				**†**													✓						
**Environmental cleaning:**																																	
Cleaning solution	✓	✓	✓	✓	✓	✓	✓	✓	✓	✓	✓	✓	✓	✓	✓	✓	✓	✓	✓	✓	✓	✓	✓	✓	✓	✓	✓	✓	✓	✓	✓	✓	✓
Terminal cleaning	✓	✓	✓	✓	✓		✓	✓	✓	✓	✓	✓	✓	✓	✓					✓	✓	✓	✓			✓			✓	✓		✓	✓
**Contamination sources:**																																	
Individual devices	✓	✓	✓	✓	✓	✓	✓	✓	✓	✓	✓	✓	✓	✓	✓	✓	✓	✓	✓	✓	✓	✓	✓		✓	✓	✓	✓	✓	✓	✓	✓	✓
Thermometers (no re-use)	✓	✓	✓	✓			✓	✓		**†**		✓		✓					✓	✓		✓	✓	**†**			✓				✓	✓	
Laundry (L)/Dishes (D)	SP	SP^a^		**†**	**†**	**†**	**†**	**†**	**†**		**†**	**†**	**†**		**†**					L:AR	D**†**		L**†**			L**†**			**†**	**†**	**†**	SP	**†**
**Education:**																																	
Staff	✓	✓	✓	✓	✓				✓		✓	✓	✓	✓	✓					✓	✓	✓	✓	✓				✓	✓	✓	✓	✓	
Patients/Visitor	✓	✓	✓	✓	✓			✓	✓	✓	✓	✓	✓	✓	✓				✓	✓	✓	✓	✓	✓		✓			✓	✓		✓	✓
**Case detection:**																																	
Test of cure	✓	✓	✓	✓		✓	✓		✓	✓	✓	✓	✓	✓					✓		✓	✓	✓			✓				✓	✓	✓	✓
No test if asymptomatic	✓	✓	✓	✓	✓		✓		✓	✓	✓	✓	✓	✓	✓	✓	✓		✓	✓	✓	✓	✓	✓					✓	✓	RNM	✓	
No testing infants	✓	**†**	✓	✓	**†**						✓	**†**	✓	✓	**†**				✓	✓^e,g^	✓	✓	✓								NA		
Diagnosis algorithm	**†**	PCR^c^ **†**2-3s^a,b,c^	**†**	**†**	**†**	**†**				**†**	**†**		2-s	**†**	2-s				2-3s	2-s^g^	**†**	2-s	2-s	**†**		2-s				2-s		**†**	
Surveillance‡	✓v	✓	✓	✓	✓m				✓v	✓	✓v	✓	✓m	✓		✓		✓v	✓	✓m	✓m	✓m	✓	✓					✓	✓	✓	✓	✓
Molecular methods	§	✓	§¶	§	§	**†**		§¶		§¶	§¶	§¶	§	§¶	§¶				**†**	§¶	§¶	§¶		**†**						**†**		§	
**Outbreak management**	✓	✓^a,b,c^	✓	✓	✓					✓	✓	✓	✓	✓				✓		✓	✓	✓	✓	✓					✓	✓		✓	

LTCF – long–term care facilities, AR – area of research, RNM – recommendation cannot be made, UI – unresolved issue, Inc – inconclusive. AICA: Australian Infection Control Association

*Documents (if multiple, per country): a: APIC Association for Professionals in Infection Control and Epidemiology (2013), b: SHEA/IDSA Society for Healthcare Epidemiology of America/Infectious Diseases Society of America (2014), c: AJG American Journal of Gastroenterology (2013), d: Position Statement (2014) e: England (2008), f: England (2013), g: England (2012), h: ASID: Australasian Society for Infectious Diseases (2011). A tick (✓) means that a recommendation is available.

**†**Information available/No detailed recommendations.

‡Some information obtained from other sources (eg, Department of Health websites) v: voluntary, m: mandatory components Diagnosis algorithm: 1–, 2–, 3–step (1–s, 2–s, 3–s): 2 or 3–s: combination of sensitive (eg, Glutamate dehydrogenase) followed by specific test (to confirm toxin: Enzyme immunoassay toxin A or A/B, polymerase chain reaction or toxigenic culture).

§,¶Molecular methods: § – outbreak, ¶ – severe cases.

**Table 2:** CDI–IPC: pharmacological agents and transmission control (patient–care related)*

	IPC area							
	**Pharmacological methods**		**Contact precautions**			**Personal protection**		**Hand hygiene**
	Antibiotic stewardship	Probiotics	Single room	Cohorting	Duration (based on cases’ diarrhea)†	Gloves	Gowns	
**Acute care – North America:**								
Canada (2013)	✓		✓	✓	R or NI	✓	✓	S/W (preferably at point–of–care or as soon as sink is available or ‡) ABHR
APIC (2013)	✓	Inconclusive	✓	✓	R>48h	✓	✓	S/W and ABHR, or ABHR (endemic)
SHEA/IDSA (2014)	(II, Basic)	UI	(III, Basic)	(Basic)	R or R>48h (Basic), if ‡: D (III Special)	(II Basic), UI	(III Basic), UI	WHO 5 moments, if ‡, S/W, ABHR
AJG (2013)	**Strong** (H)	RNM **Strong** (L)	**Strong** (H)	**Strong** (H)	R; **Strong** (H)	**Strong** (M)	**Strong** (M)	S/W **Strong** (M)
**Acute care – Europe:**								
ECDC (2008)	**IA** (1a)	RNM	**IB** (1b, 2b)	**IB** (1b, 4)	R>48h; **II** (4)	**IB** (1b, 2b)	**IB** (1a, 1b, 4)	S/W (not ABHR alone), **IB** (2a, 2b, 2c)
Austria (2007)	**IA** (1a)	Tx, R–CDI, PS	**IB** (1b, 2b)	**IB** (1b, 4)	R>48h; **II** (4)	**IB** (1b, 2b)	**IB** (1a, 1b, 4)	S/W and ABHR (limitations), **IB** (2a, 2b, 2c)
Belgium (2008)	✓		✓	✓	R>48 or 72h; **(Level 2)**	**(Level 1)**	**(Level 1)**	S/W and ABHR
Bulgaria (2009)	✓		✓	✓	R>48h	✓	✓	S/W
Cyprus (2014)	✓	RNM	✓	✓	R>48h	✓	✓	S/W, ABHR after S/W (limitations)
Denmark (2011)			✓	✓	R>48h	✓	✓	S/W, ABHR (limitations)
Finland (2007)			✓	✓	R>48h	✓	✓	S/W then ABHR (limitations)
France (2010)	✓		✓	✓	R	✓	✓	S/W then ABHR (limitations)
Germany (2009)	✓		✓	✓	R>48h	✓	✓	S/W (preferred and if soiling), ABHR
Hungary (2011)	**IA, IB, IC**	Tx, R–CDI	**IB**	**IB**	R>72h **II**	**IB**	**IB**	S/W and ABHR; **IB**
Ireland (2014)	✓	Tx, R–CDI	(C–D)	(D)	R/R>48h (D)	(A)	(D)	S/W (A)
Italy (2009)	**IA** (1a)	RNM	**IB** (1b, 2b)	**IB** (1b, 4)	R>48h **II** (4)	**IB** (1b, 2b)	**IB** (1a, 1b, 4)	S/W; not ABHR alone; **IB,** (2a, 2b, 2c)
Lithuania (2011)	✓		✓	✓	R>48h	✓	✓	S/W
Luxembourg (2007)	✓		✓		R	✓	✓	S/W then ABHR
Macedonia (2014)	✓	Tx, R–CDI	✓	✓		✓	✓	S/W, ABHR (limitations)
Netherlands (2011)	✓		✓	✓	R>48h	✓	✓	S/W, (ABHR not added value)
Romania	✓	Tx, R–CDI	✓	✓	R>48 or 72h	✓	✓	S/W, no AB–solutions
England (2008)	**B**	RNM^2013^	**B**	**B**	R>48h **C**	**A**	**A–B**	S/W then ABHR **A**
N. Ireland (2008)	✓		✓		R>72h§	✓	✓	S/W (ABHR limitations)
Scotland (2014)	**IA, IB, II**	Tx, R–CDI: insufficient evidence	**IB**	**IB**	R>48h **II**	**IB**	**IB**	S/W (not ABHR alone), **IB** (staff)**; II** (visitors)
**Acute care – Western Pacific:**								
ASID/AICA (2011)	✓	RNM^PS^	✓	✓	R>48h, if ‡:D	✓	✓	WHO 5 moments, Primary use ABHR, If soiled: S/W
Hong Kong (2014)	✓	AR	✓	✓	R or NI	✓	✓	WHO recommendations, S/W
Japan (2008)	✓	✓	✓			✓	✓	S/W
New Zealand (2013)	¶		✓		R>48 h§	✓	✓	WHO 5 moments, S/W or ABHR, if ‡: S/W and ABHR
Singapore (2013)	¶		✓	✓		✓	✓	S (antiseptic)/W or ABHR
**Acute care – South East Asia:**								
Thailand (2009)	✓	Tx, R–CDI				✓		S/W
Acute care – Latin America								
Chile (2012–13)	✓		✓	✓	R or D	✓	✓	S/W
Uruguay (2015)	✓		✓	✓	D	✓	✓	S/W
**Long term care:**								
SHEA (2002)	**A–B** (I, II, III**)**	RNM	**B** (III)	✓	R	**A** (I)	RNM	S/W or antimicrobial agent ; **B** (III)
Canada (2013)	✓		✓	✓	R or NI	✓	✓	S/W (preferably at point–of–care or where sink is available, or if ‡), ABHR
Germany (2012)			✓	✓	R>72h	✓	✓	S/W and ABHR

APIC – Association for Professionals in Infection Control and Epidemiology, SHEA – The Society for Healthcare Epidemiology of America, IDSA – Infectious Diseases Society of America, AJG – American Journal of Gastroenterology, ECDC – European Centre for Disease Prevention and Control, ASID – Australasian Society for Infectious Diseases, AICA – Australian Infection Control Association, S/W – soap/water, ABHR – alcohol–based hand rub, AR – area of research, PS – position statement, UI – Unresolved issue, R–CDI – prevention of recurrent CDI, Tx – treatment, RNM – recommendations cannot be made

*If grading of evidence available: **Strength of recommendations bold font;** (Quality of evidence). Type of documents and scope of included documents vary: eg, Denmark, Finland, and the Netherlands focus on hygiene. Table adapted from Martin et al [16]. Strong recommendations**: IA–IB, A–B, Level 1, I–II;** To be considered**: II, C, Level 3, III.** Quality of evidence grading: High**: (**1a–1c), (H), (I), (A**);** Medium–Low: **(**2a–4), (M–L), (B), (II–III), (B–C)**;** Expert opinion**:** 5; D.; Legal: IC. A tick (✓) indicates recommendation available.

†Lifting of contact precaution measures: case diarrheal status: resolved (R) or non–infectious (NI) or period (hours) after symptoms resolved, (D) Discharge.

‡Outbreak.

§Bristol Stool chart.

¶Information available/No detailed recommendation.

**Table 3:** CDI–IPC strategies for transmission control (environment), education, and case detection*

	IPC Area								
	**Environmental cleaning†**	**Medical equipment**		**Education**		**Case detection**		**Surveillance‡**	**Outbreak**
		**Patient–dedicated or single–use**	**No electronic (E) or rectal (R) thermometers**	**Staff**	**Visitors/patients**	**No test of cure**	**No testing/treating asymptomatic patients**		
**Acute care – North America:**									
Canada (2013)	S, C [1]	✓	E	✓	✓	✓	✓	✓	✓
APIC (2013)	C§	✓	E, R	✓	✓	✓	✓	✓	✓
SHEA/IDSA (2014)	S§, C (III) UI	✓	E	(III, Basic) UI	(III, Basic)	(III, Basic)	(II)	(III Basic)	✓
AJG (2013)	S§, C; **Strong** (H)	**Strong** (M)	E**; Strong** (M)	**Strong** (M)		**Strong** (M)	**Strong;** Test: (High); Tx: (Low)	**Conditional** (M)	✓
**Acute care – Europe:**									
ECDC (2008)	S, C [1]; **IB** (2b, 2c)	**IB** (1b)	E; **IA** (1b, 2b)	**IA** (1a, 2b, 4, 5)		**IA** (1a)	**IB** (2b, 3b, 4)	**IB** (2b, 3b, 4, 5)	✓
Austria (2007)	S; **IA** [1]^–PS^	**IB** (1b, 2c, 4)	R; **IA** (1b, 2b)	**IA;** (1a, 2b, 4, 5)	If¶	**IA** (1a)	**IB** (2b, 3b, 4)	**IB** (2b, 3b, 4, 5)	✓
Belgium (2008)	C [1,2]	**(Level 1–2)**		✓	✓		✓	✓	✓
Bulgaria (2009)	S, C, not alcohol	✓				✓			
Cyprus (2014)	C [1]	✓	R			✓	✓		
Denmark (2011)	C [1]	✓	R		✓				
Finland (2007)	C [1,2]	✓		✓	✓	✓	✓	✓	
France (2010)	S, C	✓	**		✓	✓	✓	✓	✓
Germany 2009)	S, C, peracetic acid	✓		✓	✓	✓	✓	✓	✓
Hungary 2011)	S, C; **IB–IC**	✓	E; **IA**	**IA**		**IA**	**IB**	**IB–IC**	✓
Ireland (2014)	C [1] (D)	✓		(D)	(C–D)	(D)	(D)	(B)	✓
Italy (2009)	C [1]; **IB** (2b, 2c)	**IB** (1b)	E,R; **IA** (1b, 2b)	**IA** (1a, 2b, 4, 5)		**IA** (1a)	**IB** (2b, 3b, 4)	**IB** (2b, 3b, 4, 5)	✓
Lithuania (2011)	C	✓		✓	✓		✓		
Luxembourg (2007)	S, C	✓					✓		
Macedonia (2014)	S, C	✓					✓		
Netherlands (2011)	C	✓						✓	✓
Romania	D, S,C [1], other††	✓	E		✓	✓	✓		
England (2008)	C [1]; **B**	**B**	E	**A–C**			**B**	Mandatory **B**	✓
N. Ireland (2008)	C or D/C [1]	✓		✓	✓	✓	✓	Mandatory	✓
Scotland (2014)	C [1]; **IB**	**IB**	E; **IA**	**IA**		**IA**	**IB**	Mandatory	✓
**Acute care – Western Pacific:**									
ASID/AICA (2011)	S, C [1], if ¶: FM, 1–step: D/S	✓	R	✓	✓	✓	✓	Min: Facility	✓
Hong Kong (2014)	✓		**	✓	✓		✓	Facility	✓
Japan (2008)	✓	✓							
N. Zealand (2013)	S, D/C [1]	✓			✓	✓			
Singapore (2013)	S, C [1]	✓	E, R						
**Acute care – South East Asia**:									
Thailand (2009)	S, C, other ††	✓		✓					
Acute care – Latin America									
Chile (2012–13)	S, C [1,2]	✓		✓	✓		✓	Facility based	✓
Uruguay (2015)	C [1,2]	✓		✓	✓	✓	✓	✓	✓
**Long term care:**									
SHEA (2002)	S, C; **B** (II)	**B** (III)	E; **A** (II)	**B** (III)		**B** (II)	Tx: **A** (I)	**B** (III)	
Canada (2013)	S, C [1]	✓	E	✓	✓	✓	✓	✓	✓
Germany (2012)	S: no alcohol; ammonium	✓			✓	✓		✓	

APIC – Association for Professionals in Infection Control and Epidemiology, SHEA – The Society for Healthcare Epidemiology of America, IDSA – Infectious Diseases Society of America, AJG – American Journal of Gastroenterology, ECDC – European Centre for Disease Prevention and Control, ASID – Australasian Society for Infectious Diseases, AICA – Australian Infection Control Association, UI – Unresolved issue

*Type of documents and scope of included documents vary: eg, guidelines from the Netherlands and Denmark focus on hygiene. Table adapted from Martin et al [16]. If grading of evidence available: **Strength of recommendations bold font;** (Quality of evidence). A tick (✓) indicates recommendation available. Strong recommendations**: IA–IB, A–B, Level 1, I–II;** to be considered**: II, C, Level 3, III.** Quality of evidence: High**: (**1a–1c), (H), (I), (A); Medium–Low*:* **(**2a–4), (M–L), (B), (II–III), (B–C)**;** Expert opinion**:** (5); (D); Legal: (IC).

**†**Cleaning solutions/methods: C: Chlorine–based; S: Sporicidal; D: Detergent; FM: Fluorescent markers. **[**Chlorine**–**based concentration] 1: at least 1000 ppm; 2: 5000 ppm

‡Some information obtained from other sources (eg, Department of Health website).

§EPA–approved.

¶Outbreak.

**Information available/No detailed recommendation.

††Alkaline glutaraldehyde, ethylene.

**Correspondence to:** Dr Harish Nair Centre for Global Health Research Usher Institute of Population Health Sciences and Informatics University of Edinburgh Medical School Teviot Place Edinburgh EH8 9AG UK Harish.Nair@ed.ac.uk

### IPC Area 1: Pharmacological methods

*Antibiotics****:*** The strong risk posed by antibiotics for CDI was mentioned in the majority of documents (**Table 1** and **Table 2**). Recommendations included: to minimise use among patients already at increased risk, stop any CDI–inciting antimicrobials such as broad–spectrum cephalosporins (3rd generation), penicillins, fluoroquinolones, and clindamycin in suspected cases [23,24,29], or promoting the implementation of antibiotic stewardship programmes (ASP). Few documents detailed the specific roles and responsibilities of different stakeholders (eg, infection control teams, administration, pharmacists, microbiologists, clinicians, and senior management). Detailed overviews of procedures recommended for establishing, implementing, and monitoring ASP in different settings were also reported [25,31,32,34–36].

*Evidence assessment:* Concordance between the evidence grades given in different guidelines was high. Guidelines strongly recommended the cautious use of antibiotics to prevent CDI and the evidence grade was awarded the highest levels.

*Discussion:* Although one guideline established that available evidence on the effect of ASP did not fully meet all criteria for the highest level of quality (research has mainly relied on before–and–after studies) [26], judicious use of antibiotics was widely recognized as essential for CDI prevention. Despite the limitations in the evidence, the beneficial effect of prudent antibiotic use on CDI is noteworthy. A recent systematic review and meta–analysis quantified the effect of both persuasive (education and guidance) and restrictive (approval required, removal) ASP for CDI [65]. A significant protective role (overall risk ratio 0.48, 95% confidence interval CI 0.38–0.62) was found, with the strongest evidence for restrictive programmes and those with the longest duration. Similarly, another review found that ASP and environmental disinfection were the two most important IPC for CDI in hospitals [18].

ASP require adequate resourcing (human and financial), thus they need to be well designed, integrated, audited, and monitored as parts of larger HAI IPC strategies [66]. Furthermore, the potential effects of utilizing antibiotics considered to be non–CDI–inciting, such as gentamicin, have been raised as important considerations to monitor [67]. Globally, an assessment of ASP showed that although strategies within programmes in 67 countries vary significantly, commonalities do exist and important challenges demand concerted worldwide action, such as the continuous prospective measurement of well–defined outcomes and appropriate resourcing [68].

*Probiotics:* Several guidelines recognized the suggested use of probiotics for the prevention of CDI. Nine documents labeled it as an *area of research* or declared *no recommendation can be made*. Others mentioned probiotics within descriptions related to CDI treatment and their potential role in preventing recurrences of CDI, but offered no formal recommendation (**Table 1** and **Table 2**).

*Evidence assessment:* One guideline [23] stated that moderate evidence existed supporting the use of two probiotics to decrease the incidence of antibiotic–associated diarrhea, but quality of evidence was low for CDI.

*Discussion:* Recently, a group of experts proposed a statement recommending utilization of two specific probiotics (*L. acidophilus* CL1285 and *L. casei* LBC80R) for CDI [69]. Although systematic reviews and meta–analyses report a protective effect of probiotics [70–72] and some publications reviewed here mention their potential use, studies exploring the contribution of probiotics to CDI prevention are largely limited by high heterogeneity between studies, high risk of bias, inadequate study power or significant levels of missing outcome data [26]. In light of these limitations in the evidence base, guidelines that systematically graded evidence stated that current scientific evidence on probiotics’ effect on CDI is insufficient to recommend their use for IPC.

*Gastric acid suppressants:* Guidance indicates that proton pump inhibitors (PPI) and histamine receptor antagonists (H_2_RA) should be considered as important risk factors for CDI but conclude that the issue remains *unresolved* with no official recommendation for CDI (**Table 1**).

### IPC Areas 2–4: Transmission based control measures – patient–care related strategies

### Isolation of cases

Isolation of CDI cases, confirmed and suspected, was widely recommended together with the use of en–suite bathrooms or individual bedpans. Guidelines also recommended cohorting CDI patients (**Table 1** and **Table 2**), if necessary. The benefits and considerations stated, beyond preventing the spread of *C. difficile* spores, included effective allocation of human and economic resources and the development of specific expertise among dedicated staff managing the isolated patient/cohort. Maintaining contact precautions until at least after diarrheal episodes have stopped (most commonly for 48 hours or longer) was generally recommended. However, extended contact precautions until the discharge of the CDI case [26,60,63] were also advised. Administrative support and communication were underscored as key factors given that isolation of cases can incur managerial difficulties and costs.

### Personal protective equipment (PPE)

Adequate use of PPE by health care workers caring for CDI cases, particularly gloves and gowns, was consistently and strongly recommended as an important precautionary measure in all documents. Use of PPE by visitors was recommended, but knowledge on the beneficial effect was labeled as an *unresolved issue* [26].

### Hand hygiene

The importance and challenges associated with effective hand hygiene in the context of *C. difficile* IPC were discussed in all documents. Special attention was drawn to limitations of disinfection hand with alcohol–based hand rubs (ABHR) as they are non–sporicidal and do not remove *C. difficile* spores from contaminated hands. Guidance on best practices varied and included the preferential use of soap and water when caring for patients with CDI, especially during outbreaks, raising awareness and warning health care providers about the limitations of ABHRs [38,48,49], or stressing the WHO hand hygiene recommendations and the primary use of ABHR to prevent confusing messages [60].

*Evidence assessment:* The reported quality of the evidence on the protective effect of isolation/cohorting and on the optimum duration of contact precautions for CDI ranged from low to high. Evidence was graded of high quality for outbreak situations, in one guideline [23].

The use of gloves and gowns was strongly recommended, but the quality of available evidence was deemed mixed.

The evidence on the effect of different hand hygiene practices was reported to be of moderate quality and the efficacy and usefulness of disinfection over hand–washing for hand hygiene purposes was reported as an *area of controversy* [26]. These differences in reporting the value of hand hygiene practices stem from research showing that hand–washing with soap and water is the most efficacious way to remove *C. difficile* spores. However, while the use of ABHR alone is not effective, its use does not appear to be detrimental in terms of impacting directly on CDI rates [73].

*Discussion:* It is noteworthy that there is a reliance on evidence from studies of multidrug–resistant organisms to prevent CDI through patient–care strategies [29] and a paucity in studies that have evaluated their efficacy during endemic periods [17,22]. Additional studies are necessary to further clarify the effects of the use of ABHR on CDI and make a more robust conclusion.

Challenges to elucidate the effect of isolation procedures as a means to prevent transmission of CDI will be influenced by each facility’s ability to detect CDI cases promptly, availability of isolation rooms, and duration of measures. Nonetheless, recent attempts have been made to provide an estimate of the effect of isolating CDI cases. For instance, a retrospective cohort study reported a 43% (95% CI 7–65%) drop in *C. difficile* acquisition rate in a facility with single–rooms in its ICU wards compared to multi–bed rooms [74]. An increased risk of recurrence (odds ratio OR: 3.77 95% CI 1.37–10.35) among previously cohorted patients has also been reported [75]. Although shedding of *C. difficile* spores and evidence of contamination after resolution of diarrhea has been found [76,77], the effect of longer isolation periods and isolation on the incidence of CDI or risk of transmission remains poorly understood.

Hand hygiene and adequate use of PPE is vital for HAI prevention. Although the use of ABHRs is inadequate to eliminate *C. difficile* spores and hand washing is preferred (a message conveyed in most guidelines), concerns exist about compliance and detrimental effects of mixed instructions for hand hygiene [73]. Recently, a study found that compliance with WHO–recommended practices by health care workers caring for patients with CDI was observed to be approximately 60–70%, with no hand hygiene conducted inside isolation rooms. A higher compliance was observed for the use of gloves (~ 85–90%) and gowns (~ 88–97%) [78]. Clearly more research is needed, especially for the effect of different hand hygiene practices on CDI incidence during endemic periods [22], but a stronger emphasis on the use of gloves has been underscored as an important, economical, and potentially more effective measure to prevent *C. difficile* transmission [79].

### IPC Area 5–6: transmission control – environmental contamination

All documents addressed the importance of environmental cleaning to prevent *C. difficile* transmission. Chlorine–based and sporicidal agents were the most commonly recommended solutions. The use of other technologies, including UV light or hydrogen peroxide vapor, was discussed and highlighted as an *unresolved issue* [80,81]. The vast majority of guidelines advised that medical equipment used for CDI cases should be patient–dedicated or disposable, where possible. Commonly reported potential sources of contamination included items that come into direct contact with patients (blood pressure cuffs, stethoscopes, thermometers) or are at risk of contamination due to soiling (beds, furniture, sinks, floor, curtains, etc.). Thorough cleaning of all equipment used after caring for CDI cases or that entered the isolation/cohort room (including dishes and laundry) was also advised. Recommendations explicitly addressing the role of electronic or rectal thermometers were identified in 17 documents (**Table 1**, **Table 2** and **Table 3**).

*Evidence assessment:* Despite the high level of agreement across guidelines on the use of sporicidal chlorine–based solutions, the optimum type of solution used for environmental cleaning of *C. difficile* was considered to remain as an *area of controversy* [26]. The strongly recommended use of patient–dedicated or of single–use devices was common and guidelines concurred that currently available evidence is of moderate quality (individual randomly–controlled trials and non–randomized studies). The quality of evidence in support of replacement of electronic for single–use disposable thermometers was graded as high/moderate quality.

*Discussion:* Published data on the effect of environmental decontamination with solutions currently recommended to prevent CDI transmission *“have not been consistent”* and the effect of bleach has only been demonstrated in outbreak situations [82] and in combination with other IPC measures. Additionally, concerns about their corrosive properties and potentially harmful effect on the health and safety of staff need to be weighed carefully against the benefits of their use [83]. Beyond the physical environment, attention has been drawn to other potential sources of contamination. For instance, whole genome sequence–based studies have the potential to clarify issues about patient–to–patient transmission including the role of asymptomatic *C. difficile* colonised patients, but more research is needed [77].

### IPC Area 7: education of staff and patients/visitors

Education was defined as instructions, information, training, educational campaigns or workshops for health care facility workers, patients, or visitors on any aspect of CDI–IPC. Over half of the guidelines recommended an education component for education of staff (health care, cleaning, or auxiliary personnel), patients and/or visitors (**Table 1**, **Table 2** and **Table 3**).

*Evidence assessment:* Education was strongly recommended across guidelines, with the quality of evidence for its effect being graded high to low.

*Discussion:* The effect of educational programmes as CDI–IPC interventions has not been fully assessed. However, studies have reported a worrying gap in the knowledge about CDI among health care workers [84–86] and the suboptimal quality of educational materials for patients [87]. Lack of clinical suspicion was identified as a key factor leading to under– and misdiagnosis of CDI cases in Europe [15], which can hinder adequate and timely implementation of IPC measures.

### IPC Area 8–9: case detection and surveillance

Surveillance was recommended at various levels in guidelines: from national, including LTCF/NH [25,31,32] to at least facility–based level with a minimum of hospital–onset health care–associated cases [26,62]. Documents providing information on surveillance recommended the use of standardised case definitions. Most guidelines included a statement or clarifications that discouraged conducting *test of cure*. Over half of the guidelines explicitly recommended against testing or treating asymptomatic patients (**Table 3**).

Regarding testing policies and laboratory assays, the use of standardised criteria (eg, Bristol stool chart [25,31,38]) or definitions (≥3 unformed stools in ≤24 consecutive hours [26]) was reported to identify adequate samples to be tested. However, other documents described, generally, the importance of testing “unformed/diarrheal stools”. Additional strategies included no testing of infants (mainly in Europe), no (or limited) repeat testing. General descriptions were also identified for the use of molecular typing methods for severe cases or during outbreaks. **Table 1** includes information on case detections methods in documents reviewed. Notably, guidelines reported toxin enzyme immunoassays as not suitable as stand–alone, molecular tests were strongly recommended as standard test in the US [23], and multi–step algorithms were generally described in other documents.

*Evidence assessment*: The quality of evidence to not conduct a test of cure after CDI’s symptoms resolution was awarded the highest score in Europe, but moderate and low scores in recent guidelines by US–based organizations [23,26]. Guidelines agree that, currently, there is no evidence to support the detection or routine screening of *C. difficile* among asymptomatic patients, with published studies being of moderate and low quality. The strength of recommendation for CDI–targeted surveillance systems ranged from strong to conditional, and of legal character. Mandatory or legal components regarding reporting of cases were described for the UK, Ireland, and Hungary, compared to recommended laboratory–based sentinel and facility–based voluntary systems in other countries.

*Discussion:* Prompt case detection is vital for the implementation of IPC strategies for CDI. Concerted efforts to better understand and address the burden of CDI, such as for the development of case definitions for surveillance [88] and improved understanding of laboratory tests’ limitations [89] and diagnosis procedures have been promoted since the mid–2000s.

The use and implications of differential CDI case detection methods have been described in recent studies [6,15,90,91]. In Europe, it was shown that testing policies varied widely, a factor that contributed to a large number of CDI cases being missed on a daily basis. A notable exception was the UK, where both under– and misdiagnosis is uncommon, as national guidelines have been introduced to standardise laboratory diagnosis including confirmatory procedures [15,43]. In the US, 43% of 120 laboratories surveyed used molecular assays as first– or second–line for diagnosis of CDI, similarly to the percentage using enzyme immunoassay tests alone (42%) [91]. In this survey, use of molecular tests was more likely to be accompanied by higher rejection rates for unnecessary testing (ie, formed stools, test for cure, or duplicates within 7 days). Although faster molecular methods have the potential to reduce isolation costs and treatment delays as compared to multi–step algorithms, the unknown proportion of cases diagnosed by high–sensitive molecular tools who may not be CDI cases needs careful consideration as inconsistent results have been found on the impact confirmatory procedures can have on clinical practice [89,92]. False positives can lead to unnecessary implementation of IPC measures and treatment (which in itself increases the risk of developing CDI due antibiotic use) and distort the epidemiological picture of burden of disease.

### IPC Area 10: outbreak management

Over half of documents included a labeled and separate section for recommendations during outbreaks or periods of increased incidence [23-25,28–30,32–37,44,45,49,54,60,62,63]. Case definitions of outbreaks were not clearly reported in most guidelines. Surveillance systems were recognized as an essential tool to identify and monitor outbreaks [26]. Two formats for case definition of CDI outbreaks were identified:

- Definitions based on exceeding triggers based on local CDI epidemiology (hospital or ward’s, as available) [25,34,35] with the addition of a specified period of time criteria [29] (eg, expected incidence of CDI exceeded for 1 [62] or 2 [32,63] weeks in a specific area).
- Defined thresholds and criteria (eg, 3 or more cases of hospital–acquired CDI for 2 weeks in a specific area [44]; 2 or more cases caused by the same strain over a defined period and related in time and place [31]).

IPC recommendations in different guidelines for outbreaks ranged in detail and depth but most convey a common message: during CDI outbreaks, all IPC measures should be enhanced. Additional key recommendations during outbreaks included:

- Promoting timely communication between healthcare workers and other infection prevention and control efforts.
- Assessing antibiotic prescribing and environmental cleaning practices to prevent further use of high–risk CDI antibiotics and ensure high quality control of decontamination.
- Collecting samples for molecular typing of CDI cases to determine if outbreak is associated with hyper–virulent strains (eg, 027, 176, or 078) **(****Table 1****)**.
- As resources and logistics allow, setting up dedicated administrative systems to manage admissions and staff to CDI–affected wards.

Documents lacking a clear section for IPC of outbreaks, drew attention to specific strategies by differentiating best practices during outbreaks as compared to endemic periods (eg, environmental cleaning – increase frequency [52] or hand washing practices [58] – consider restricting hand hygiene to handwashing with soap and water) (**Table 1**, **Table 2** and **Table 3**).

### Prevention of CDI and the need for coordinated strategies

Implementation of general HAI IPC strategies is crucial to minimise risk of CDI and, as this review shows, several targeted efforts for *C. difficile* exist. Furthermore, clear and consistent guidance is needed to integrate CDI prevention efforts into larger HAI–control programmes effectively. We reviewed documents with CDI–IPC recommendations in 28 countries and found a general consensus on a selected number of strongly recommended strategies: prudent use of antimicrobials, adequate environmental cleaning with agents with sporicidal effects, time–sensitive isolation, and barrier methods for staff including gowns and gloves. However, we also some noted important variations.

Differences in availability of strategies in documents were found, which can be explained by differences in the scope and type of documents, the recognized CDI burden, health care systems infrastructures, and national legislation requirements. However, varying or imprecise guidance suggests that there is still room for further primary studies but also greater harmonisation of CDI–IPC guidelines, namely in the assessment of the quality of the evidence. For instance, clear recommendations on most accurate laboratory algorithms can be provided rather than descriptions of available methods. Such guidance has the potential to promote best and standardised practices but also raise awareness of the limitations of the alternatives and inform allocation resource for IPC. Optimum CDI case detection methods are changing and updated guidelines will soon become available [93]. Due to the systematic methods used to develop guidelines by professional bodies, such as ECDC and SHEA/IDSA, these are important resources from which to draw information for establishing or updating national guidance and achieve a greater international alignment, yet allowing for national matters to be taken into consideration.

In light of previous widespread of *C. difficile* hyper–virulent strains, a clear section with detailed measures for endemic and epidemic periods should be available in guidelines, to address the burden of CDI effectively. We found a general absence of such distinction in half of the documents, as well as differential appraisal of the quality of evidence for key strategies (eg, the effect of isolation during epidemics was graded mixed to high). Our review underlines previous findings of a lack of uniformity in the assessment of evidence in guidelines [16] and suggests a need for stronger international alignment of CDI–IPC guidance guided by an objective assessment of the literature.

Agreement about best practices across guidelines has been indispensable for advancing efforts in an integrated manner on the role of antibiotics, which could also enable coordinated efforts in other areas. The CDC’s recent recommendations for both acute health facilities [66] and LTCFs [94] are significant resources informed by CDI–IPC efforts. Future studies on the effect of the introduction of ASP and close monitoring of the effect of previously considered “low risk” antibiotics are required to continue informing our understanding of antibiotics and CDI. It is imperative that coordinated efforts are undertaken to elucidate strengths and weaknesses of the evidence base and update guidance and convey clear CDI–IPC statements for other areas. For instance, beneficial effects of probiotics for CDI are not supported by high quality studies, as described previously. We identified consensus on the recommendation from guidelines with systematic assessment of the literature, but also ambiguous guidance in descriptive documents. *C. difficile’s* epidemiology continues to evolve and review of guidelines by qualified local professional bodies is necessary to recommend best practices, based on the strongest quality of research. Such exercises have the potential to support the development of context–appropriate tools for different stakeholders, such as checklists, cleaning regimes, or education packages for health care workers (an example [95]), cleaning staff, and patients.

The paucity of guidelines pertinent to different types of health care settings is concerning due to the increased incidence of CDI in the last 20 years. It is also of concern that the overall effect of interventions in high risk settings such as LTCFs is under–recognized, where suboptimal compliance to recommendations has previously been identified [37], where *C. difficile* is a common pathogen causing diarrhea [96], and where over–prescription of antibiotics is prevalent. In the USA, over 4 million Americans reside in LTCFs and a substantial majority (70%) are at increased risk of CDI due to high use of antibiotics in this setting (40–75% prescribed incorrectly) [94]. It is important to adapt guidance based on acute care settings experiences with evidence from interventions in LTCF and nursing homes [96,97]. We recommend high quality studies on the effect of IPC strategies in nursing homes and LTCFs are synthesized, appraised, and as possible, incorporated into guidelines to inform targeted IPC of CDI efforts.

Beyond single and targeted strategies, organizational accountability, mentioned in few of the guidelines, demands particular attention as it is essential for development of country–specific implementation of strategies. Stakeholders’ roles and responsibilities, including that of governments and senior management staff, need to be clear and informed by evidence relevant to national structures. For instance, in the UK, investigations on significant recent outbreaks have resulted in reports with recommendations which inform the roles of responsibilities of care and management staff [98,99]. Recognition of gaps in the system enabled development of new guidance, such as procedures to capture *C. difficile*–associated deaths, and detailed methods to strengthen coordination of IPC teams.

### Bundles for prevention and control of CDI

Available evidence indicates that multi–faceted programmes of CDI prevention have the potential to be substantially effective and cost–saving. In the UK, CDI–IPC strategies include legislative support (ie, mandatory national surveillance systems and wider organizational accountability, including defined roles and responsibilities for all groups of health care staff and senior management), hand and environmental hygiene campaigns, and optimised testing/diagnosis techniques [7]. In addition to cost savings, quality improvement in health care and patient safety are also major priorities. Based on this comprehensive IPC approach, the estimated cost reduction associated with a decrease in the number of CDI cases (5–15%) ranged from GBP 4.65–13.94 million [100]. In the United States, a recent study estimated that if basic recommendations by the SHEA/IDSA were introduced nationally, over 5 million CDI cases among patients 65 years of age or older would be averted during a 5–year period. This reduction in number of cases would result in US$ 2.5 billion of savings [19]. Of note, this study adopted a conservative economic model which estimated the cost of isolation until discharge, rather than until symptom resolution.

### Emerging topics and the need for more research

**Box 1** presents a summary of research questions as identified in the documents reviewed. The need for innovative prevention technologies and more effective cleaning solutions were discussed. High quality studies on the effect of interventions such as the use of case notification systems, on the potential roles of different health care workers in detection of cases and implementation of IPC, and on unresolved issues were recognized as important areas of research.

Box 1 **CDI prevention and infection control emerging topics and future steps**

**Area of controversy**

• Ability of diluted sodium hypochlorite or other sporicidal agents used for environmental decontamination [26].

• Reliance on alcohol–based hand hygiene products [26].

• Management, including detection or isolation, of patients colonized (asymptomatic) with *C. difficile* without CDI history [26,45].

**Unresolved issues (UI) or other (O) strategies identified in guidelines**

Case detection, including roles of different health care workers in CDI–IPC

Notification systems or laboratory–based alert systems

O: Role of community pharmacists [25], medical equipment and health care staff in ambulances [31,58].

UI: Alert for changes in the rate, complications, or severity of CDI that may indicate the introduction of new strains [29] or for cases readmitted or transferred) [26,45].

UI: Role of nurses (standing orders or nurse–driven protocols) [26].

**Transmission control**

O: Use of bleach or cleaning wipes for disinfection or Fluorescent markers or adenosine triphosphate to measure organic material [25,34–36].

O: Development of protocols for disinfection of equipment and environment and monitoring [34,35].

O: Visitor and staff management: visitors/staff with diarrhea should not visit patients in the hospital [45].

O: Facility design (eg, selection of materials for surfaces, adequate number of hand washing facilities) [34,35].

UI: Use of gown and gloves by visitors [26].

UI: Use of soap that contains antiseptic substances [45].

UI: No–touch disinfection technologies as component of IPC strategies (UV, hydrogen peroxide vapor) [26].

**Pharmacological agents**

UI: Use of Vaccines and immunotherapies [32].

UI: Role of probiotics as primary prophylaxis [26].

UI: Restriction of gastric acid suppressants [25,26].

**Education**

UI: On–going assessment of CDI knowledge and intensified CDI education among health care and cleaning personnel [26].

**LTCF Research questions and relevant issues**

Notification of CDI among LTCF residents to relevant staff in the acute care setting if transfer is necessary [25,58].

Attention to CDI cases’ activities and placement (shared rooms) [35].

Monitoring compliance with infection prevention and control guidance and adequate implementation of strategies (including diarrheal, outbreaks, and waste management and access to laboratory services) [37].

**The following research questions [**27**]**

• Are older patients truly at increased risk of acquiring *C. difficile* or CDI? If so, what determinants are responsible?

• Are therapeutic strategies equally effective in older population and in younger adults?

• Are differences between risks for CDI outbreaks explained by variations in antibiotic exposure or are there other factors?

• What are the variables that influence transmission of *C. difficile* between residents in long–term care settings?; What is the role of environment, and patient care practices?

• What level of environmental cleaning, hand hygiene, or glove use is optimal to limit transmission of the organism?

• Are infection control recommendations different for patients with diarrhoea compared with those without?

Notably, there is a need for higher quality and comparable evidence on the attributable effects of existing CDI prevention measures, especially during endemic periods [17,18,22]. Adequate surveillance and improved detection of cases require critical attention, as our review found that differences in approaches exist. Although best practices are still a matter of debate, well–established, resourced, and audited surveillance systems for CDI are essential. Surveillance supported by consistent, clear, and cost–effective laboratory testing practices (including rejection policies) has the potential to inform the effect of CDI–targeted IPC and novel interventions, such as “bundles” or vaccines. Costs associated with implementation of effective surveillance and case detection methods should be assessed in light of the benefits for patients’ safety and care. Further, adequate reporting of aspects of infectious control measures is needed in future studies to identify optimum CDI control programmes (eg, dedicated personnel time, laboratory supplies, and outbreak investigations) [18]. We echo previous recommendations that future studies should adhere to the ORION statement to be able to synthesize evidence in a more transparent and consistent manner [15,18,19], thus support greater harmonisations of CDI–targeted IPC efforts.

Understanding the prophylactic effects of pharmacological methods is an area of great interest for CDI–IPC. Passive immunization to toxins TcdA and TcdB has been tested for the prevention of recurrences. Given its high cost and transient protection, active immunization is currently viewed as a potentially more cost–effective strategy. Both toxoid–based and peptide vaccines are currently under development [101,102]. Another developing area of research is the prevention of recurrent episodes and severe disease outcomes with more effective antibiotics. Recently, a 3–4 fold decrease in CDI recurrence and 28–day mortality was observed in hospitals with routine use of fidaxomicin as first–line treatment, and at a greater rate than in hospitals with selective use of this antibiotic [103]. As burden of disease associated with CDI remains high, cost–effective pharmacological methods to prevent incident, recurrent, or severe outcomes represent a key area for targeted IPC.

### Limitations

The present review has limitations. We relied on electronic search methods of publicly available documents. We also relied on translation to examine the full text of several guidelines and one document could not be translated [51]. However, we identified and reviewed a large number of documents obtained through comprehensive searches undertaken by two reviewers. While the interpretation of some of the guidelines’ through our review may be influenced by language restrictions, the majority of documents included in analysis are in languages that reviewers manage fluently. Finally, we did not review compliance with national guidelines, treatment of CDI, or strategies not within the 10 selected IPC areas as it was beyond the scope of this review.

## CONCLUSIONS

Our review findings indicate a widespread awareness of the importance of CDI–IPC guidelines but there are significant gaps which still exist. The review identified published guidelines from regions which have experienced an increase in the incidence of CDI in recent years (such as the USA, Canada, Europe and the Western Pacific) and also countries where epidemiology of *C. difficile* has not been extensively examined (such as Thailand, Chile, and Uruguay). However, we did not retrieve IPC guidelines for CDI from several countries in South America, South East Asia, and Europe and none from Africa and Eastern Mediterranean. We reviewed documents for Bulgaria, Hungary, Macedonia, Poland, and Romania, which were not included in a previous assessment of European guidelines. Our review also found only a few clear and specific recommendations for LTCFs and nursing homes, mainly from North America, Europe and Western Pacific. This represents a large gap in an important global infection control area. Thus, this review adds to the existing collection of IPC guidance availability for *C. difficile* [16,104] and provides a global overview of approaches and challenges for those interested in developing or revising protocols for CDI prevention and control.

This review of guidelines also highlights the need for greater international harmonisation in the assessment of the evidence underpinning IPC recommendations for CDI and for more research. Key strategies strongly and consistently recommended in published guidelines included: ASP, environmental and medical devices cleaning, use of protective equipment (gloves and gowns), and prompt isolation of CDI cases. Surveillance and education were also strongly recommended. High quality research, other than for high–risk antibiotics, is still needed. Our review shows that much of the evidence underpinning the guidance was graded of medium to low level, by the use of 4 different ranking schemes (assessed only in guidelines from the USA and Europe) and different primary studies were considered in different guidelines. The recommended establishment of surveillance and standardised monitoring systems will help develop comparable studies and better evaluate the effect of interventions on CDI incidence in the future.

Our review of unresolved issues and inconsistently identified strategies indicates that implementation of CDI–IPC measures variations between world regions exist, mainly for hand hygiene and case detection approaches (including laboratory testing policies). Country–specific organizational accountability roles require key attention for successful IPC efforts and control outbreaks associated with *C. difficile*. Strategies on the use of probiotics, gastric acid suppressants, and on the potential roles of IPC stakeholders could benefit from clear recommendations statements. Studies that provide more robust estimates of interventions’ effects in high–risk settings such as LTCF and of emerging IPC technologies, such as vaccines, have the potential to inform coordinated efforts and advise priority setting exercises.
