# Supplementary material for: Infection prevention and control of Clostridium difficile: a global review of guidelines, strategies, and recommendations
Source: J Glob Health. 2016 Nov 23;6(2):020410. doi: 10.7189/jogh.06.020410 (PMC5140074; doi:10.7189/jogh.06.020410)
Supplement: Online Supplementary Document [file jogh-06-020410-s001.pdf]

Online Supplementary Document

Balsells et al. Infection prevention and control of Clostridium difficile: a global review of guidelines, strategies, and recommendations

J Glob Health 2016;6:020410

Table S1. Summary of ranking systems used for grading evidence in CDI-IPC guidelines

**Quality of Evidence**							
**Guideline**	**Ranking system**	**High**	**Medium**	**Low or Expert opinion**			**Legal requirement**
**ECDC 2008**<br>Austria 2007<br>Italy 2009	Oxford Centre for Evidence-Based Medicine	**1a:** SR (homogeneity) of RCTs<br>**1b:** Individual RCT (narrow CI)<br>**1c:** Studies outcome 'All/none'	**2a:** SR (homogeneity) of cohort<br>**2b:** Individual cohort study (including low-quality RCTs)<br>**2c:** 'Outcomes' research; ecological studies	**3a:** SR (homogeneity) of case–control studies<br>**3b:** Individual case–control study	**4:** Case series (and poor quality cohort and case–control studies)	**5:** Based on Expert opinion without explicit critical appraisal, physiology, bench research, 'first principle'	
**Ireland 2014**	Consensus grade	**A:** Meta-analysis/SR of RCTs or from at least one RCT	**B:** One controlled trial without randomisation (e.g. cohort study), a quasi-experimental study, or extrapolated from RCT	**C:** Comparative, correlation, case control studies or, extrapolated from A or B.		**D:** Expert committees, reports or opinions, the clinical experience of respected authorities, and the conclusions of the Development Group	Legal requirement
**SHEA/IDSA 2014**<br>**AJG 2013**	GRADE	**I: High** - further research is unlikely to change confidence in current estimate of effect<br>* Several studies with no major limitations and little variation<br>* CI summary estimate: narrow	**II:** Moderate - further research is likely to have an important impact<br>* Few studies and some have limitations but not major flaws, some variation in-between<br>* CI summary estimate: wide	**III: Low** - further research very likely to change the estimate<br>* Studies have major flaws and important variation in-between studies.<br>* CI summary estimate: very wide, or there are no rigorous studies, only expert consensus			
**SHEA/IDSA 2002 (LTCF)**	*	**I:** Good evidence	II Moderate evidence	**III:** Poor evidence			
**Strength of Recommendation**							
**Guideline**	**Ranking system**	**Strongly recommended**	**Strongly recommended**	**Recommended**	**Suggested/To be considered**	**Unresolved**	**Legal requirement**
**ECDC 2008**<br>Austria 2007<br>Italy 2009<br>Hungary 2011<br>Scotland 2014	Healthcare Infection Control Practices Advisory Committee	**IA:** Well-designed experimental, clinical, or epidemiological studies	**IB:** Some experimental, clinical or epidemiological studies and a strong theoretical rationale		**II:** Suggestive clinical or epidemiological studies or theoretical rationale	Practices for which insufficient evidence exists or no consensus regarding efficacy exists (no recommendation)	**IC:** As mandated by federal and/ or state regulation or standard (may vary among different areas)
**England 2008**	Consensus grade	**A:** SRs or individuals RCT	**B:** Non-RCT studies and/or by clinical governance reports and/or the Code		**C:** group consensus and/or strong theoretical rationale		Legal requirement
**SHEA/IDSA 2002 (LTCF)**	*	**A:** Evidence from at least one properly RCT	**B:** at least one well-designed CT without randomisation (cohort or case-controlled; preferably from more than one center), from multiple time-series studies, or from dramatic results in uncontrolled experiments		**C**: opinions of respected authorities, based on clinical experience, descriptive studies, or reports of expert committees		
**Belgium**	Simple grading	**Level 1**		**Level 2**	**Level 3**		
**AJG 2013**	GRADE	**Strong:** Evidence shows the benefit of the intervention or treatment clearly outweighs any risk		**Conditional:** Uncertainty exists about the risk-benefit ratio			

SHEA: The Society for Healthcare Epidemiology of America, **IDSA:** Infectious Diseases Society of America, **AJG:** American Journal of Gastroenterology, **ECDC:** European Centre for Disease Prevention and Control. LTCF: Long term care facilities ASID 2011 includes Quality Assessment of selected key areas as reported in Cohen et al 2010 (Recommendations for Clinical Practice of *C. difficile*) but does not grade their own guidelines, *SHEA/IDSA 2002 uses classification scheme from 1994; SR: systematic reviews, RCT: randomised controlled trials

Table S2. Main characteristics of documents with CDI-IPC guidance included in the review

Country	Year (1st issue)	Healthcare Settings	Guideline type (N/PO)	Systematic Review	Quality of Evidence Scoring	Strength of recommendations :	Language	Scope (Updates)
**North America**								
**APIC**	2013 [36] (2008)	AC, LTCF	PO	x			English	Dx, IPC, Tx
**SHEA/IDSA**	2014 [26] (2008)	AC	PO	x	x		English	Dx, IPC
	2002[27]	LTCF	PO		x	x	English	Dx, IPC, Tx
**AJG**	2013[23]	AC	PO		x	x	English	Dx, IPC outbreaks, Tx
**Canada**	2013[34]	AC	N				English	Dx, IPC
	2013[35]	LTCF	N				English	Dx, IPC
**Europe**								
**ECDC**	2008[29]	Nosocomial CDAD	PO	x	x	x	English	Dx, IPC
**Austria**	2007[24]	AC, RC	N	x*	x*	x*	German	Dx, IPC, Tx
	2014[38]	NR	N				German	Dx, IPC, Tx Position statement
**Belgium***	2008[30]	AC, RC	N			x*	French	Dx, IPC
**Bulgaria**	2009[53]	HCFs	N				Bulgarian	Dx, IPC
**Cyprus**	2014[52]	HCFs	N				Greek	Dx, IPC
**Denmark***	2011[46]	HCFs	N				Danish	Hygiene IPC
**Finland***	2007[39]	Hospitals	PO				Finnish	Hygiene IPC
**France**	2010[45]	HCFs	N				French	IPC
**Germany**	2009[54]	AC, NH	PO				German	Dx, IPC
	2012[47]	NH, Rehab, OP-Therapy	PO				German	IPC
**Hungary**	2011[33]	HCFs	N	x*		x*	Hungarian	Dx, IPC
**Ireland**	2014[25]	AC, LTCF, GP	N		x		English	Dx, IPC
**Italy**	2009[28]	HCFs	N/PO	x*	x*	x*	Italian	Dx, IPC
**Lithuania**	2011[40]	HCFs	PO				Lithuanian	Dx, IPC
**Luxembourg**	2007[41]	HCFs	N				French	IPC
**Macedonia**	2014[48]	HCFs	N				Macedonian	IPC
**Netherlands**	2011 [49] (2006)	Hospitals	N				Dutch	Hygiene
**Poland‡**	2011[51]		N				Polish	Dx, IPC, Tx
**Romania**	NY**[50]**	HCFs	N				Romanian	Dx, IPC, Tx
**England and Wales**	2008[31] (1994)	AC, CH	N			x	English	Dx (2012), IPC, Tx, (2013)
	2010[37]	CH	N				English	Tx
**Northern Ireland**	2008[44]	NR	N				English	Dx, IPC
**Scotland**	2014[32]	AC, CH	N	x*		x*	English	Dx, IPC, Tx
**Western Pacific**								
**ASID & AICA**	2011[59]	AC, RCHF	PO				English	Dx, Tx, Rec. prev
	2011[60]		PO				English	Dx, IPC Position statement
**Hong Kong**	2014[56]	HCF, RCHE	N				English	Dx, IPC
**Japan**	2008[55]	NR	N				Japanese	Dx, Tx, IPC
**New Zealand**	2013[58]	HCF , AR-RC	N				English	Dx, IPC
**Singapore**	2013[57]	AC, ILTC	N				English	Dx, IPC
**South East Asia**								
**Thailand**	2009[64]	NR	PO				Thai	Dx, Tx, IPC
**Latin America**								
**Chile**	2013, [62] 2012[61]	AC	N				Spanish	IPC
**Uruguay**	2015[63]	HCFs	N				Spanish	Dx, IPC, Tx

Notes: APIC: Association for Professionals in Infection Control and Epidemiology, **SHEA:** The Society for Healthcare Epidemiology of America, **IDSA:** Infectious Diseases Society of America, **AJG:** American Journal of Gastroenterology, **ECDC:** European Centre for Disease Prevention and Control, **ASID:** Australasian Society for Infectious Diseases, **AICA:** Australian Infection Control Association; * Previously reported as based on ECDC guidelines (Martin et al 2014); LTCF: long term care facilities, ILTC: intermediate and long-term care; RC(HE): residential care (homes for elderly); NR: not reported; AR-RC: age-related residential care; N: National agency; PO: Professional organisation; Dx: Diagnosis; Tx, treatment; IPC: Prevention and Infection Control; NY: No year; ‡ Unable to review full text
